# Construction of a high-density genetic map based on large-scale markers developed by specific length amplified fragment sequencing (SLAF-seq) and its application to QTL analysis for isoflavone content in *Glycine max*

**DOI:** 10.1186/1471-2164-15-1086

**Published:** 2014-12-10

**Authors:** Bin Li, Ling Tian, Jingying Zhang, Long Huang, Fenxia Han, Shurong Yan, Lianzheng Wang, Hongkun Zheng, Junming Sun

**Affiliations:** The National Key Facility for Crop Gene Resources and Genetic Improvement, NFCRI, MOA Key Laboratory of Soybean Biology (Beijing), Institute of Crop Science, Chinese Academy of Agricultural Sciences, 12 Zhongguancun South Street, Beijing, 100081 China; Biomarker Technologies Corporation, Beijing, 101300 China

**Keywords:** High-density genetic map, Isoflavone content, QTL, SLAF-seq, Soybean [*Glycine max* (L.) Merr.]

## Abstract

**Background:**

Quantitative trait locus (QTL) mapping is an efficient approach to discover the genetic architecture underlying complex quantitative traits. However, the low density of molecular markers in genetic maps has limited the efficiency and accuracy of QTL mapping. In this study, specific length amplified fragment sequencing (SLAF-seq), a new high-throughput strategy for large-scale SNP discovery and genotyping based on next generation sequencing (NGS), was employed to construct a high-density soybean genetic map using recombinant inbred lines (RILs, Luheidou2 × Nanhuizao, F_5:8_). With this map, the consistent QTLs for isoflavone content across various environments were identified.

**Results:**

In total, 23 Gb of data containing 87,604,858 pair-end reads were obtained. The average coverage for each SLAF marker was 11.20-fold for the female parent, 12.51-fold for the male parent, and an average of 3.98-fold for individual RILs. Among the 116,216 high-quality SLAFs obtained, 9,948 were polymorphic. The final map consisted of 5,785 SLAFs on 20 linkage groups (LGs) and spanned 2,255.18 cM in genome size with an average distance of 0.43 cM between adjacent markers. Comparative genomic analysis revealed a relatively high collinearity of 20 LGs with the soybean reference genome. Based on this map, 41 QTLs were identified that contributed to the isoflavone content. The high efficiency and accuracy of this map were evidenced by the discovery of genes encoding isoflavone biosynthetic enzymes within these loci. Moreover, 11 of these 41 QTLs (including six novel loci) were associated with isoflavone content across multiple environments. One of them, *qIF20-2*, contributed to a majority of isoflavone components across various environments and explained a high amount of phenotypic variance (8.7% - 35.3%). This represents a novel major QTL underlying isoflavone content across various environments in soybean.

**Conclusions:**

Herein, we reported a high-density genetic map for soybean. This map exhibited high resolution and accuracy. It will facilitate the identification of genes and QTLs underlying essential agronomic traits in soybean. The novel major QTL for isoflavone content is useful not only for further study on the genetic basis of isoflavone accumulation, but also for marker-assisted selection (MAS) in soybean breeding in the future.

**Electronic supplementary material:**

The online version of this article (doi:10.1186/1471-2164-15-1086) contains supplementary material, which is available to authorized users.

## Background

Soybean [*Glycine max* (L.) Merrill] is one of the most important grain legumes. It represented 57% of world oilseed production in 2012 and provides large amounts of vegetable protein [1]. In addition, soybean is the natural source of some anticancer substances such as isoflavones and lunasin [2, 3]. Many essential agronomic and quality traits have been studied through developing genetic linkage map and identifying genes or quantitative trait loci (QTLs) underlying these traits to improve yield, nutritional quality, as well as biotic and abiotic stress tolerance [4–8].

Soybean genome mapping based on molecular markers started in the early 1990s and a number of genetic maps have been constructed [9–12]. With these genetic maps, more than one thousand QTLs associated with essential traits have been identified [13]. However, most of these maps are low-density genetic maps based on low-throughput molecular markers such as restriction fragment length polymorphism (RFLP), amplified fragment length polymorphism (AFLP), and simple sequence repeat (SSR) markers. The low density of molecular markers has limited the efficiency and accuracy of QTL mapping. Previous studies have demonstrated that increasing marker density can significantly improve the resolution of a genetic map in a given mapping population [9, 12, 14]. Additionally, the development of high-throughput sequencing technology provides the capacity for developing massive single nucleotide polymorphism (SNP) markers [15]. Therefore, it is feasible to construct high-density genetic maps based on SNP markers and thereby improve the efficiency and accuracy of gene or QTL mapping. As a result, a composite genetic map, the soybean Consensus Map 4.0, was constructed with 5,500 markers by using five mapping populations of soybean [11]. Meanwhile, a 1,536 universal soy linkage panel was developed for QTL mapping [11]. Recently, a specific-locus amplified fragment sequencing (SLAF-seq) technology has been developed which exhibits advantages in high-throughput SNP marker discovery and genotyping for genetic map construction [12]. This technology created a balance between higher genotyping accuracy and relatively lower sequencing cost. It is therefore highly suitable for genetic association studies [16–18].

Isoflavones belong to a group of secondary metabolites derived from the phenylpropanoid pathway and are mainly produced in legumes. These compounds function in various biological processes in leguminous plants. For instance, they play an important role as precursors of major phytoalexins during plant-microbe interaction [19, 20]. They also serve as signal molecules during the induction of nodulation-related genes [21–23]. In addition, due to their structural similarity with estrogen, isoflavones have aroused lots of attention in recent years for their association with human health. Studies have shown that these compounds have positive effects on decreasing risk of breast cancer, menopausal symptoms, osteoporosis, dementia and cardiovascular disease [24–31].

Due to their important roles for plants and humans, studies on accumulation of isoflavones have been performed worldwide [32–36]. The ultimate goal of these studies is to illustrate the genetic basis of the isoflavone accumulation and to develop a series of cultivars with varying isoflavone content. Because isoflavone content is a typical quantitative trait influenced by both genetic and environmental factors, the identification of major or minor QTLs underlying isoflavone content over various environments have been conducted, and these loci were mapped to almost all chromosomes of soybean [12, 37–43]. Despite extensive studies, major consistent QTLs underlying isoflavone content across various environments remain unidentified.

In this study, the SLAF-seq was used in whole-genome genotyping for soybean recombinant inbred lines (RILs) and a high-density genetic map was constructed based on the developed SLAF markers. The characters of this genetic map are analyzed and discussed in detail in this study. To our knowledge, this map is the densest to date among individual soybean genetic maps, and it exhibited high resolution and accuracy. Moreover, the QTLs underlying the isoflavone content were identified and analyzed based on this map.

## Results

### Genotyping of RIL population based on SLAF-seq

The RIL population was genotyped by using SLAF-seq technology. According to the results of pilot experiment, *EcoR*I and *Mse*I were chosen for the SLAF library construction. The library consisted of SLAF fragments that were 500-550 bp in size. High-throughput sequencing of this library was performed subsequently. In total, 23 Gb of data containing 87,604,858 pair-end reads were generated using the Illumina Genome Analyzer IIx. Of the high-quality data, ~144 Mb were from the female parent with 1,815,592 reads and ~166 Mb were from the male parent with 2,091,373 reads. The number of reads for the 110 RILs ranged from 140,896 to 1,016,448 with an average of 564,973.

**Figure 1:**
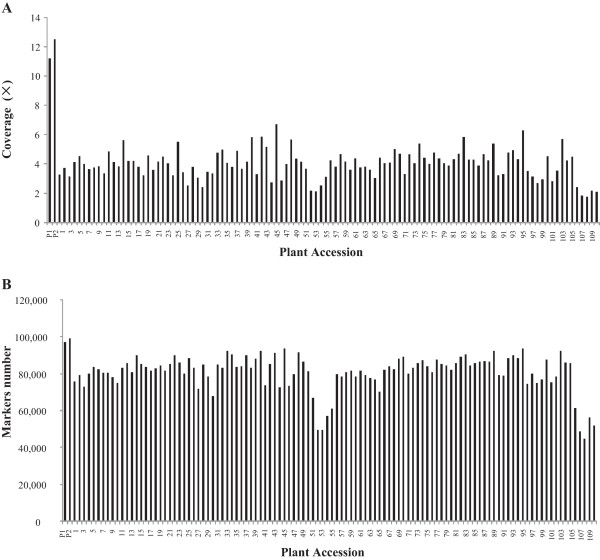
**Coverage and number of markers for each RIL and their parents. A** Coverage of SLAF markers. **B** Number of SLAF markers. The x-axes in A and B indicate the plant accession including the female parent (Luheidou2, designated as P1) and the male parent (Nanhuizao, designated as P2) followed by each of the RILs. The y-axis in A indicates the coverage of markers (fold) and the y-axis in B indicates the number of markers.

The reads were then mapped to the reference soybean genome (*cv.* Williams 82), and the reads which could be mapped to a single locus were considered as effective SLAFs. In this study, 80% of the reads could be exactly mapped to specific chromosome regions. The numbers of SLAFs in the female and male parents were 97,016 and 99,229, respectively. The numbers of reads for SLAFs were 1,085,756 and 1,241,835 in the female and male parents, respectively. Thus, the average coverage for each SLAF marker was 11.20-fold for the female parent and 12.51-fold for the male parent. For the RIL population, the number of SLAF markers ranged from 44,626 to 93,583 with an average of 80,567. The number of reads for SLAFs ranged from 78,488 to 629,831 with an average of 328,862. The coverage ranged from 1.76-fold to 6.73-fold with an average of 3.98-fold (Figure 1).

Among the 116,216 SLAFs obtained, 9,948 were polymorphic. The polymorphic rate of these SLAFs was 8.6 % (Table 1), which is consistent with a previous study [44]. The SLAFs were further screened to filter out markers unsuitable for genetic map construction. Finally, a total of 5,785 SLAFs were used for the high-density linkage map construction. All of these markers were SNP-type markers. Approximately 2.1% of genotyping data were missing in the RIL population for these 5,785 SLAFs. Therefore, the integrity of SLAF markers for the RIL population was 97.9%.

**Table 1:** **SLAF marker mining results**

	Number of SLAF markers	Ratio
**Polymorphisms**	9,948	8.6%
**Non-polymorphisms**	106,268	91.4%
**Total**	116,216	100.0%

### High-density genetic map construction for soybean using SLAF-seq genotyping data

**Figure 2:**
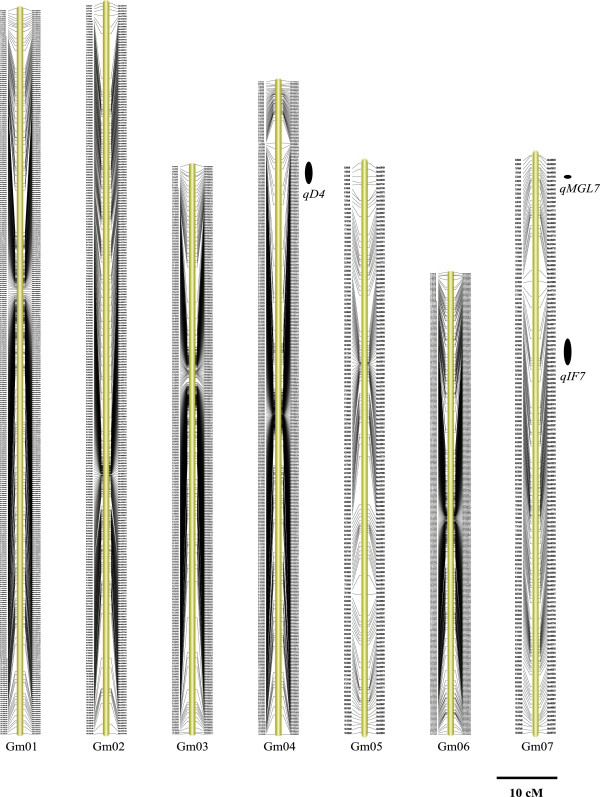
**The soybean high-density genetic map: Linkage groups Gm01-Gm07.** The SLAF markers and their location are shown on the right and left side, respectively. The 11 QTLs identified across various environments are depicted on the right side of each linkage group as black ovals. The name of each QTL, shown near each oval, is a composite of the influenced trait: genistin (G), daidzin (D), glycitin (GL), malonyldaidzin (MD), malonylgenistin (MG), malonylglycitin (MGL) and total isoflavone content (TOT) followed by the chromosome number. For QTLs underlying the contents of multiple isoflavone components, the name is a composite of isoflavones (IF) followed by the chromosome number.

**Figure 3:**
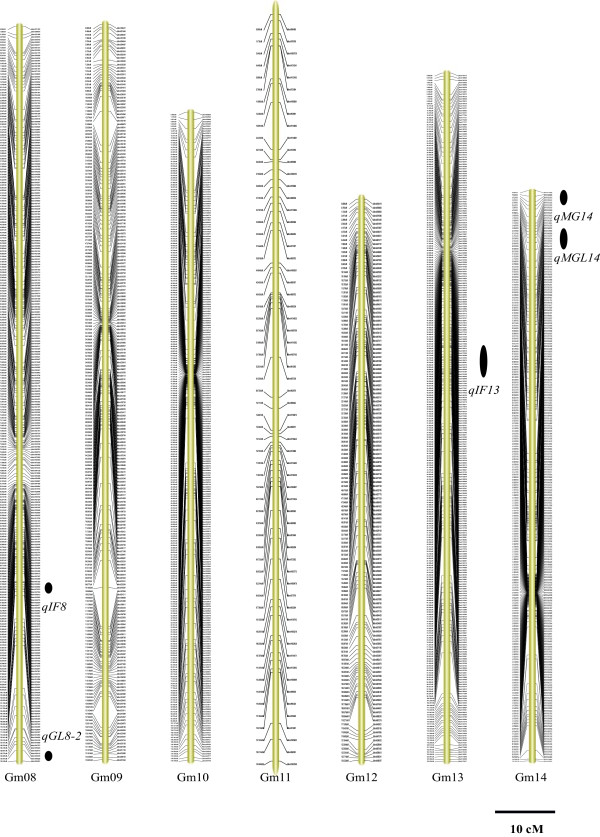
**The soybean high-density genetic map: Linkage groups Gm08-Gm14.** The SLAF markers and their location are shown on the right and left side, respectively. The 11 QTLs identified across various environments are depicted on the right side of each linkage group as black ovals. The name of each QTL, shown near each oval, is a composite of the influenced trait: genistin (G), daidzin (D), glycitin (GL), malonyldaidzin (MD), malonylgenistin (MG), malonylglycitin (MGL) and total isoflavone content (TOT) followed by the chromosome number. For QTLs underlying the contents of multiple isoflavone components, the name is a composite of isoflavones (IF) followed by the chromosome number.

**Figure 4:**
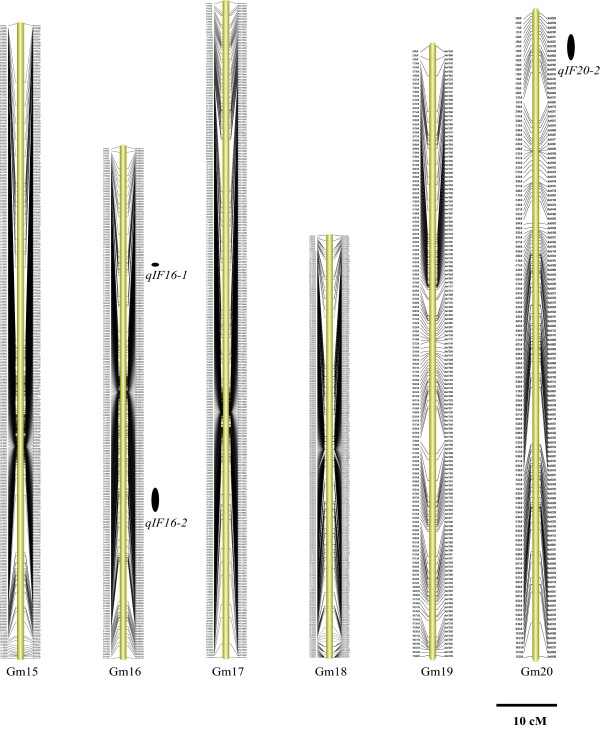
**The soybean high-density genetic map: Linkage groups Gm15-Gm20.** The SLAF markers and their location are shown on the right and left side, respectively. The 11 QTLs identified across various environments are depicted on the right side of each linkage group as black ovals. The name of each QTL, shown near each oval, is a composite of the influenced trait: genistin (G), daidzin (D), glycitin (GL), malonyldaidzin (MD), malonylgenistin (MG), malonylglycitin (MGL) and total isoflavone content (TOT) followed by the chromosome number. For QTLs underlying the contents of multiple isoflavone components, the name is a composite of isoflavones (IF) followed by the chromosome number.

**Figure 5:**
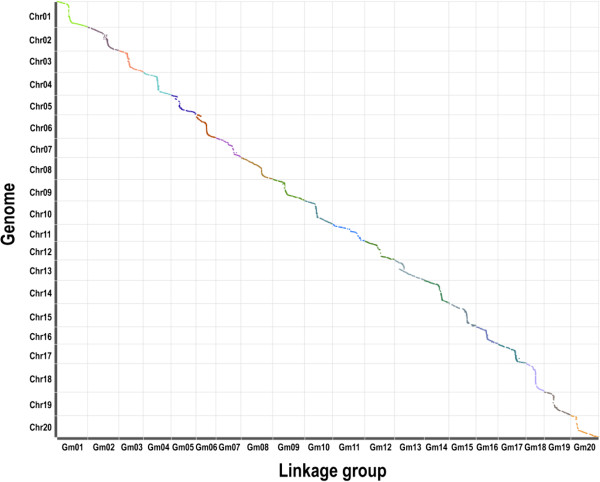
**The collinearity plot of 20 LGs with the soybean reference genome.** The x-axis indicates linearity order of linkage map and the y-axis demonstrates the linearity order of physical position in the soybean reference genome. All 5,785 SLAF markers are plotted as a scatter diagram. Different colors indicate different chromosomes or LGs.

A high-density genetic map was constructed by using the SLAF-seq genotyping data. The 5,785 SLAF markers were grouped into 20 linkage groups (LGs) and the order of these markers was arranged (Figures 2, 3 and 4). The total genetic distance of this map was 2255.18 cM. The average distance between adjacent markers was 0.43 cM. The largest LG was Gm11 with 62 SLAF markers and a length of 134.05 cM. The smallest LG was Gm18 with 647 SLAF markers and a length of 74.90 cM. The mean chromosome region length was 112.76 cM. We also found that approximately 90.8% of the intervals between adjacent markers were less than 1 cM. There were a total of 40 gaps that were 5 to 10 cM in length and four gaps of > 10 cM. The largest gap was mapped to Gm15 with 13.06 cM in length. The detail characters of these 20 LGs are shown in Table 2. To assess the quality of this genetic map, two dimensional heat maps of the 20 LGs were generated separately by using pair-wise recombination values for the 5,785 SLAF markers. A heat map for Gm20 is shown as an example in Additional file 1: Figure S1. These heat maps indicated that the construction of this map was accurate, as the recombination frequency was considerably low among adjacent markers. The collinearity of each LG with the soybean reference genome was also analyzed. As shown in Table 2 and Figure 5, a relatively high collinearity was observed between 20 LGs and the reference genome. An example collinearity map for Gm20 is also shown in Additional file 1: Figure S2.

**Table 2:** **Description of characteristics of the 20 LGs in the high-density genetic map**

LG	Size (Mb)	No. of SLAFs	distance (cM)	Average distance between markers (cM)	Collinearity %	Largest Gap(cM)	Gap < 5 cM	Kb/cM
Gm01	55.91	359	129.04	0.35	62.0%	7.56	99%	433.28
Gm02	51.65	281	130.36	0.46	64.0%	7.97	99%	396.21
Gm03	47.78	356	101.25	0.28	54.0%	6.42	99%	471.90
Gm04	49.24	448	116.16	0.25	54.0%	7.89	99%	423.90
Gm05	41.93	138	103.10	0.74	68.0%	8.74	98%	406.69
Gm06	50.72	379	82.14	0.21	58.0%	3.29	100%	617.48
Gm07	44.68	137	104.48	0.76	77.0%	6.28	99%	427.64
Gm08	46.99	278	132.34	0.47	69.0%	7.73	99%	355.07
Gm09	46.84	222	132.88	0.59	68.0%	7.00	98%	352.50
Gm10	50.96	277	116.80	0.42	61.0%	8.08	99%	436.30
Gm11	39.17	62	134.05	2.16	85.0%	9.12	90%	292.20
Gm12	40.11	139	124.22	0.89	74.0%	7.33	98%	322.89
Gm13	44.40	295	123.83	0.41	65.0%	7.38	99%	358.56
Gm14	49.71	348	102.40	0.29	59.0%	5.90	99%	485.45
Gm15	50.93	414	112.84	0.27	57.0%	13.06	99%	451.35
Gm16	37.39	310	91.18	0.29	60.0%	6.71	99%	410.07
Gm17	41.90	407	116.60	0.28	60.0%	9.26	99%	359.35
Gm18	62.30	647	74.90	0.11	53.0%	8.61	99%	831.78
Gm19	50.58	150	110.08	0.73	66.0%	8.04	98%	459.48
Gm20	46.77	138	116.53	0.84	76.0%	10.33	97%	401.36
Maximum	62.30	647	134.05	2.16	85.0%	13.06	100%	464.75
Minimum	37.39	62	74.90	0.11	53.0%	3.29	90%	499.20
Total	949.96	5,785	2,255.18	0.43	64.5%	/	/	421.23

### The determination of isoflavone components in soybean seeds

The determination and quantification of 12 isoflavone components for 110 RILs and their parents were performed in this study. Consistent with our previous study [45], six major isoflavone components including daidzin, glycitin, genistin, malonyldaidzin, malonylglycitin, and malonylgenistin, were detected in soybean seeds. The precise contents of these six isoflavone components were quantified separately. Other components were not quantified due to the very low concentrations. The total isoflavone content was designated as the sum of these six major isoflavone components.

**Figure 6:**
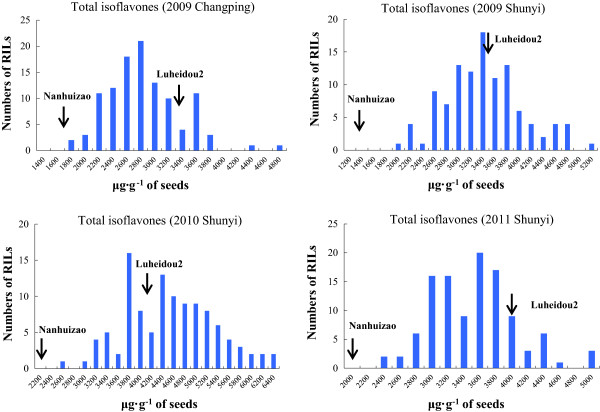
**Frequency distribution of total isoflavone contents for the 110 RILs.** The arrows indicate total isoflavone contents for two parents of the RILs (*cv*. Luheidou 2 and Nanhuizao).

The frequency distributions of total isoflavone content for the 110 RILs were also analyzed over four environments. As shown in Figure 6, the two parents differ significantly in total isoflavone content over four environments. The *cv.* Luheidou2 exhibited a higher mean value of total isoflavone content over four environments (3,697 μg · g^−1^), while the *cv.* Nanhuizao displayed a lower value of 1,816 μg · g^−1^. The total isoflavone content of individual RILs ranged from 1,729 to 6,223 μg · g^−1^ and exhibited in a typical quantitative manner. Moreover, a considerable transgressive segregation was found in the RIL population (Figure 6), which indicates a pyramiding of independent loci for total isoflavone content from both parents. Similar distributions were observed for individual isoflavone components (Additional file 1: Figure S3).

The analysis of variance (ANOVA) for isoflavone content was performed over four environments to detect the sources of phenotypic variation. As the Table 3 shows, the phenotypic variations for individual and total isoflavone contents were significantly influenced by both genetic and environmental factors. On the other hand, correlation analyses indicated that the total isoflavone contents were significantly correlated among the four environments (Additional file 1: Table S1), suggesting an important role of genetic factor in isoflavone accumulation. Due to the environmental effect on isoflavone accumulation, the QTLs for isoflavone content were identified separately in each environment. The four environments (2009 at Changping, 2009 at Shunyi, 2010 at Shunyi, and 2011 at Shunyi) were designated as E1-E4. The common loci detected across multiple environments were considered as consistent QTLs for isoflavone content.

**Table 3:** **ANOVA of individual and total isoflavone contents for 110 RILs over four environments**

Trait	Mean ± SD (μg · g ^−1^ )	Sources of variation					
		Genotype			Environment		
		Mean square	F	***P*** value	Mean square	F	***P*** value
Daidzin	224.91 ± 86.20	10256.93	8.23	< 0.0001	578864.76	464.53	< 0.0001
Glycitin	64.53 ± 18.66	706.77	7.06	<0.0001	14337.06	143.22	< 0.0001
Genistin	321.02 ± 153.06	17441.38	4.78	<0.0001	2396631.36	656.37	< 0.0001
Malonyldaidzin	1262.69 ± 288.63	221868.23	10.74	<0.0001	1876993.38	90.84	< 0.0001
Malonylglycitin	153.64 ± 51.85	4060.10	1.89	<0.0001	12171.21	5.68	< 0.0001
Malonylgenistin	1450.70 ± 389.79	185623.87	5.81	<0.0001	12008730.73	376.14	< 0.0001
Total isoflavones	3500.32 ± 869.90	1157236.90	8.17	<0.0001	53251596.80	376.00	< 0.0001

### Data analysis and QTL mapping

**Figure 7:**
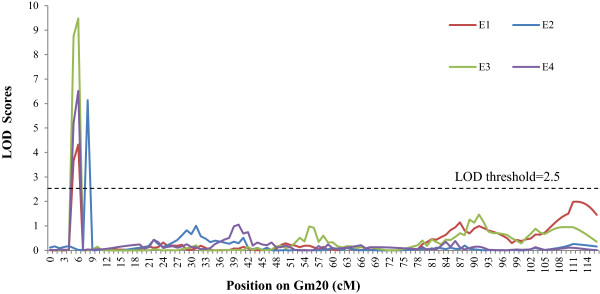
**LOD curves on Gm20 for daidzin among four environments.** The curves indicate LOD scores of SLAF markers against their genetic position on Gm20. Different colours represent different environments (E1-E4). Red curve indicates the LOD curve in E1 (i.e. Changping in 2009), blue curve represents LOD curve in E2 (i.e. Shunyi in 2009), green curve indicates the LOD curve in E3 (i.e. Shunyi in 2010), and purple curve represents the LOD curve in E4 (i.e. Shunyi in 2011). The dashed line indicates the threshold LOD score.

Based on the high-density genetic map, the QTLs underlying the isoflavone content were identified. The threshold of logarithm of odds (LOD) scores for evaluating the statistical significance of QTL effects was determined using 1,000 permutations. As a result, intervals with a LOD value above 2.5 were detected as effective QTLs using the QTL ICIMapping V3.3 software. According to the threshold, 89 QTLs were detected for individual and total isoflavone contents. The 89 QTLs overlapped and can be classified into 41 loci (shown in Additional file 1: Table S2). Of these loci, 11 QTLs were detected across various environments (shown in Table 4, Figures 2, 3 and 4 and Additional file 1: Figure S4). These QTLs may represent the genetic basis of the isoflavone accumulation and were thus focused in our subsequent analysis. As described in Table 4, the 11 QTLs were mapped to Gm4, Gm7, Gm8, Gm13, Gm14, Gm16 and Gm20. The average phenotypic variance explained by individual QTL of the 11 loci ranged from 8.61% to 28.00% and the average LOD score ranged from 3.50 to 12.40. The additive effects of *qIF8*, *qD4*, *qIF7*, *qGL8-2*, *qMGL14*, *qMG14* and *qIF20-2* were derived mainly from the female parent (*cv.* Luheidou2), while those of *qMGL7*, *qIF13*, *qIF16-1* and *qIF16-2* were derived mainly from the male parent (*cv.* Nanhuizao). Our comparative analysis showed that 18 of the total 41 loci overlapped with previously reported QTLs (Additional file 1: Table S2). For the 11 consistent QTLs, five loci (*qD4*, *qIF7*, *qGL18-2*, *qIF13* and *qIF16-1*) overlapped with previously reported QTLs (Additional file 1: Table S2). The remaining six QTLs (*qMGL7*, *qIF8*, *qMGL14*, *qMG14*, *qIF16-2* and *qIF20-2*) were not found in previous reports, thus they may present novel loci for the isoflavone content. Noticeably, *qIF20-2* was associated with daidzin, genistin, malonyldaidzin, malonylgenistin, and total isoflavone content across multiple environments. The LOD score of this locus ranged from 2.86 to 18.89 for individual isoflavone components among various environments, and it could explain 8.67% to 35.29% of phenotypic variance (Table 5). Therefore, this QTL may be the major locus for isoflavone accumulation. As an example, the LOD curves of *qIF20* for daidzin were shown in Figure 7.

**Table 4:** **The characters of 11 QTLs associated with individual and total isoflavone contents across various environments**

Name ^1^	Effect ^2^	Chr ^3^	Interval ^4^	IC ^5^	LOD ^6^	PVE ^7^	ADD ^8^
*qD4*	D-E3,E4	4	M634469-M625652	14.67-18.75	6.98	16.00	23.40
*qIF7*	G-E2,E3; MD-E2,E3; MG-E2,E3; TOT-E2,E3	7	M519691-M491368	33.21-38.13	8.17	14.30	137.00
*qMGL7*	MGL-E2,E3	7	M523495-M518225	3.62-4.24	4.59	13.30	−23.00
*qGL8-2*	GL-E2,E4	8	M1060220-M1053451	130.48-132.34	12.40	28.00	8.98
*qIF8*	MG-E1,E4; TOT-E4	8	M1045556-M1053803	101.44-101.76	5.73	16.90	144.00
*qIF13*	D-E2,E3,E4; G-E3; MG-E3; MD-E4	13	M153314-M163112	45.77-51.16	4.91	12.50	−44.00
*qMGL14*	MGL-E1,E3	14	M542764-M545951	6.83-10.60	4.00	8.61	9.96
*qMG14*	MG-E1,E3	14	M575406-M531913	0.00-2.57	3.50	9.54	83.10
*qIF16-1*	D-E2,E3; TOT-E2	16	M889900-M897989	20.85-21.19	3.96	8.56	−49.00
*qIF16-2*	MD-E1,E3,E4;D-E4	16	M895322-M899870	59.53-63.53	5.12	15.10	−79.00
*qIF20-2*	D-E1,E2,E3,E4; G-E2,E3,E4; MD-E1,E2,E3, E4;MG-E2,E3; TOT-E1,E2,E3	20	M943408-M941848	3.83-8.43	9.11	19.62	117.00

^1^The name of QTL, is a composite of the influenced trait: genistin (G), daidzin (D), glycitin (GL), malonyldaidzin (MD), malonylgenistin (MG), malonylglycitin (MGL) and total isoflavones (TOT) followed by the chromosome number. For QTLs underlying the contents of multiple isoflavone components, the name is a composite of isoflavones (IF) followed by the chromosome number.

^2^The Effect of QTL is composite of the particular isoflavone component followed by the specific environments. It represents the particular isoflavone components [i. e., genistin (G), daidzin (D), glycitin (GL), malonyldaidzin (MD), malonylgenistin (MG), malonylglycitin (MGL)] and total isoflavones (TOT) that are controlled by this QTL in specific environments [i. e., E1 (2009 at Changping), E2 (2009 at Shunyi), E3 (2010 at Shunyi), and E4 (2011 at Shunyi)].

^3^Chr indicates chromosome.

^4^Interval indicates confidence interval between two SLAF markers.

^5^IC indicates the interval of confidence in centimorgan.

^6^LOD indicates the logarithm of odds score.

^7^PVE indicates the phenotypic variance explained by individual QTL.

^8^ADD indicates the additive effect value.

The LOD scores, PVE values, and additive effect values are shown as mean values for QTLs with multiple effects.

**Table 5:** **Additive effect of** ***qIF20-2*** **for individual and total isoflavone contents across various environments**

Name ^1^	Effect ^2^	Interval ^3^	IC ^4^	LOD ^5^	PVE ^6^	ADD ^7^
*qIF20-2*	D-E1	M934616-M970087	5.92-6.63	4.33	15.16	22.27
	D-E2	M947267-M941848	7.63-8.43	6.14	15.60	24.72
	D-E3	M934616-M970087	5.92-6.63	9.47	21.13	34.72
	D-E4	M934616-M970087	5.92-6.63	6.51	15.38	14.25
	G-E2	M947267-M941848	7.63-8.43	9.63	21.96	31.59
	G-E3	M934616-M970087	5.92-6.63	11.08	24.19	63.09
	G-E4	M947267-M941848	7.63-8.43	5.00	12.28	24.91
	MD-E1	M943408-M968883	3.83-4.64	4.93	17.89	104.92
	MD-E2	M934616-M970087	5.92-6.63	9.36	23.35	152.44
	MD-E3	M934616-M970087	5.92-6.63	9.08	20.13	125.71
	MD-E4	M934616-M970087	5.92-6.63	4.52	12.55	75.15
	MG-E2	M934616-M970087	5.92-6.63	18.00	27.89	129.48
	MG-E3	M968883-M934616	4.64-5.92	9.64	23.41	156.97
	TOT-E1	M934616-M970087	5.92-6.63	2.86	8.67	151.54
	TOT-E2	M934616-M970087	5.92-6.63	16.25	19.04	280.27
	TOT-E3	M968883-M934616	4.64-5.92	18.89	35.29	472.78

^1^The name of QTL, is a composite of the influenced trait: genistin (G), daidzin (D), glycitin (GL), malonyldaidzin (MD), malonylgenistin (MG), malonylgenistin (MGL) and total isoflavones (TOT) followed by the chromosome number. For QTLs underlying the contents of multiple isoflavone components, the name is a composite of isoflavones (IF) followed by the chromosome number.

^2^The Effect of QTL is composite of the particular isoflavone component followed by the specific environments. It represents the particular isoflavone components [i. e., genistin (G), daidzin (D), glycitin (GL), malonyldaidzin (MD), malonylgenistin (MG), malonylglycitin (MGL)] and total isoflavones (TOT) that are controlled by this QTL in specific environments [i. e., E1 (2009 Changping), E2 (2009 Shunyi), E3 (2010 Shunyi), and E4 (2011 Shunyi)].

^3^Interval indicates confidence intervals between two SLAF markers.

^4^IC indicates the interval of confidence in centimorgan.

^5^LOD indicates the logarithm of odds score.

^6^PVE indicates the phenotypic variance explained by individual QTL.

^7^ADD indicates the additive effect value.

To validate the QTL mapping results, the genome interval regions within these 41 QTLs were also compared with the soybean reference genome sequences. Potential genes within the QTL intervals were annotated and analyzed. As depicted in Additional file 1: Table S2, 13 genes encoding enzymes involved in the isoflavone biosynthetic pathway were discovered including *4-coumarate: coenzyme A ligase* (*4CL*), *chalcone isomerase* (*CHI*), *chalcone reductase* (*CHR*), *isoflavone synthase* (*IFS*) and *O-methyltransferase* (*OMT*). Moreover, of these 13 genes, three genes were identified within the 11 consistent QTLs. The inclusion of these genes suggests a high efficiency and accuracy of this map in QTL mapping for isoflavone content.

## Discussion

QTL mapping has been used as an efficient approach to analyze quantitative traits in plants. However, the quality of genetic maps has a significant effect on the accuracy of QTL mapping. It has been reported that increasing marker density can improve the resolution of genetic maps [12, 14]. Nevertheless, the linkage disequilibrium (LD) of soybean is significantly higher than other plants [46]. This implies a limitation on the effectiveness of increasing marker density to improve the resolution of soybean genetic maps. Therefore, a suitable marker density in genetic maps is necessary for effective QTL mapping and MAS. Because the average LD of cultivated soybean is approximately 150 kb [46], a genetic map could be theoretically saturated with 6,300 evenly distributed markers.

With the completion of the whole genome sequencing of soybean *cv.* Williams 82 and the rapid development in sequencing technology, high polymorphic single nucleotide polymorphism (SNP) markers are beginning to be used in soybean for large-scale genotyping and high-density genetic map construction [47]. The SNP markers are efficient for high-density genetic map construction because of their high-throughput compared to AFLP, RFLP and SSR markers. In this study, a SLAF-seq was used for large-scale marker discovery and genotyping to develop a high-density genetic map. The SLAF-seq has several positive characteristics such as high efficiency for marker development, low cost, and high capacity for large population [16]. By using this approach, a total of 9,948 polymorphic SLAF markers were developed, and 5,785 SNP markers were ultimately integrated into a genetic linkage map. The average genetic distance between adjacent markers was only 0.43 cM. To our knowledge, this map has the highest marker-density to date among individual experimental soybean genetic maps. Surprisingly, Gm18 has the highest number of SLAF markers, yet its linkage distance is the smallest. This may be caused by the relatively small size of our RIL population for genotyping. In this case, the recombinant events were insufficient and the distance of this LG may be underestimated.

Recently, Hyten et al. developed a high-density integrated genetic linkage map for soybean, the Consensus Map 4.0, by compositing the SNP loci data obtained from five mapping populations [11]. That map consisted of 5,500 markers and spanned a genomic map distance of 2,296.4 cM. The mean chromosome length was 114.8 cM, with a mean genetic distance of 0.6 cM between adjacent markers [11]. Compared to the soybean Consensus Map 4.0, our genetic map had similar distance in genome size but more markers, thus the mean genetic distance between adjacent markers was narrowed to 0.43 cM. Owing to the high density, the resolution of the genetic map could be improved significantly. As a result, the accuracy of QTL or gene mapping could also be improved. Moreover, our high-density genetic map was constructed based on a single RIL population, thus QTL mapping could be performed efficiently and conveniently for a given phenotypic trait (e.g., isoflavone content). In this study, amounts of reported QTLs for isoflavone content were detected, and the confidence intervals of these QTLs were almost narrowed down significantly (Additional file 1: Table S2). For instance, *qMG5*, a QTL underlying malonylgenistin, was narrowed to a 0.12 cM of interval as compared to a previously reported 3.7 cM [12]. Another QTL, *qGL5* was also mapped to a 0.36 cM region that was mapped to a 3.2 cM of interval previously [12]. The consistent major QTLs contributing to individual and total isoflavone contents across various environments, *qIF7*, *qIF9*, *qIF16-1*, and *qIF17-2* were also narrowed to 4.92, 7.35, 0.34 and 6.18 cM from previously reported 14.4, 16.2, 16.0 and 13.2 cM, respectively [37, 38, 40, 48]. In total, roughly half of the 41 QTLs were mapped to intervals within 1 cM, and the narrowest interval spanned only 0.11 cM (Additional file 1: Table S2). According to the above results, this high density genetic map exhibited higher efficiency and accuracy for QTL mapping compared to previous genetic maps. In this case, appropriate markers closely associated with isoflavone content could be easily developed for MAS.

In map-based cloning, secondary mapping populations are usually needed after primary mapping. However, development of secondary mapping populations is laborious and time-consuming work. With high-density genetic maps and the high-quality genome sequences of *cv.* Williams 82, one can directly predict candidate genes within a narrow region between two adjacent markers. The prediction, however, should depend on a high genomic collinearity of this region between the genetic map and the soybean reference genome. For this reason, the collinearity of the 20 LGs with the soybean reference genome was also determined. Our results demonstrated a relatively high collinearity between the genetic map and the reference genome (Figure 5 and Table 2). This implies the feasibility of identifying candidate genes through comparative mapping.

The isoflavone content is a typical quantitative trait that is influenced by genetic and environmental factors [12]. The bell-shaped distribution frequency in this study confirmed that this trait inherited in a quantitative manner (Figure 6). Moreover, although the parents of RILs exhibited significant difference in isoflavone content, an obviously transgressive segregation was observed within the RIL population (Figure 6), which suggests that both parents bear positive-effect alleles. This conclusion was also supported by the identification of positive QTLs from both parents (Additional file 1: Table S2). Of the 41 identified QTLs, 26 were inherited from the female parent (*cv.* Luheidou2), while 15 were inherited from the male parent (*cv.* Nanhuizao). This finding is essential for enhancing isoflavone content through genetic engineering. Interestingly, an obvious difference in transgression segregation is observed among individual isoflavone components in the RIL population. There were transgressive segregations towards both high and low isoflavone contents for daidzin and malonylglycitin. For other isoflavone components, there were transgressive segregations towards low isoflavone content for genistin and glycitin, whereas transgressive segregations towards high isoflavone content were observed for malonyldaidzin and malonylgenistin (Additional file 1: Figure S3). Due to the high proportion of the latter two isoflavone components, the total isoflavone content showed a transgression towards high isoflavone content in the RIL population (Figure 6, upper left panel). Since both parents of the RIL population bear positive-effect alleles for isoflavone content, we speculate that the difference in transgressive segregation may be caused by various gene-gene interactions inherited from the two parents of the RIL population.

Previous studies have investigated the QTLs underlying isoflavone content in soybean [12, 37–43]. Approximately 45 QTLs have been identified. In this study, 41 QTLs were identified for individual and total isoflavone contents (Additional file 1: Table S2), and approximately half of these loci have previously been associated with isoflavone content (Additional file 1: Table S2). Moreover, many genes encoding isoflavone biosynthetic enzymes were identified within the genomic region of these loci (Additional file 1: Table S2). For example, both isoflavone synthase (IFS) genes (*IFS1* and *IFS2*) that are active in soybean were found within the *qGL7* and *qIF13* regions, respectively. The IFS catalyzes the first committed step in the isoflavone biosynthetic pathway, which is a branch of the phenylpropanoid pathway. In this pathway, IFS redirects the intermediates from the flavonoid pathway to corresponding isoflavones and thus plays a key role in isoflavone biosynthesis [49]. The discovery of previously reported QTLs and genes encoding isoflavone biosynthetic enzymes suggests that this genetic map has a high resolution and accuracy for QTL mapping.

Comparative genomic analysis showed that five of these 11 consistent QTLs had been reported in previous studies, while the other six QTLs were identified for the first time. Interestingly, one of these six novel QTLs, *qIF 20-2*, had a significant effect on almost all isoflavone components (daidzin, genistin, malonylgenistin, and malonyldaidzin) and total isoflavone contents across various environments. The average phenotypic variance explained by this QTL ranged from 8.67% to 35.29 % with an average of 19.62%, and the LOD values ranged from 2.86 to 18.89, with an average of 9.11. A previous study reported another major QTLon Gm05, which also had a significant influence on individual and total isoflavone contents and could explain over 30.0% of phenotypic variance of isoflavone content [7]. These results demonstrate that despite the strong effect of environmental factors on the accumulation of isoflavones, there major QTLs or genes for isoflavone accumulation may exist.

Although the previously reported major QTL on Gm05 was also identified in our study in E2 environment (Shunyi, 2009), the phenotypic variance explained by this QTL was only 7.1%. Additionally, the *qIF20-2* has not been detected previously. That might be explained by the difference in genetic background between the parents of the two mapping populations. This explanation suggests that RIL population should be developed as much as possible from distantly related parents with significantly different genetic backgrounds, and that multiple mapping populations should be used in QTL mapping for a given trait.

Since the isoflavone content was influenced by both genotypic and environmental factors, the 11 consistent QTLs identified across various environments may bear important genes for the accumulation and regulation of isoflavones. In this study, only three genes encoding isoflavone biosynthetic enzymes were discovered within the 11 QTLs. Moreover, none of these genes was found within the novel major loci *qIF20-2*, which explained a large amount of phenotypic variance for isoflavone content. This implies that although the isoflavone biosynthetic enzymes play an important role in the accumulation of isoflavones, other unknown genes may also participate in the regulation of isoflavone accumulation. The discovery of these genes will help elucidate the mechanism of isoflavone accumulation and regulation, and provide new insights for the enhancement of isoflavone content through genetic engineering or MAS.

## Conclusions

Herein we report a high-density genetic map for soybean, which is constructed based on large-scale markers developed by specific length amplified fragment sequencing (SLAF-seq). Our results suggest that this high-density genetic map is accurate and efficient for QTL mapping. By using this map, we identified a novel major QTL underlying individual and total isoflavone contents across various environments. The high phenotypic variance explained by this locus demonstrates the importance of this locus in soybean isoflavone accumulation. Additionally, the locus may be an ideal candidate target for MAS in soybean isoflavone breeding. The genes within this locus will be studied in detail to identify the genetic architecture underlying isoflavone accumulation in our subsequent studies.

## Methods

### Plant materials and DNA extraction

We utilized a mapping population that included 200 F_5:7-8_ RILs derived from a cross between soybean *cv.* Luheidou2 (distributed in the Huang-Huai-Hai valley region of China) and *cv.* Nanhuizao (distributed in the south region of China). Both Luheidou2 and Nanhuizao are cultivar soybeans with black seed coats. However, the seed isoflavone content differs significantly. Luheidou2 exhibits high isoflavone content while the isoflavone content of Nanhuizao is relatively low. These 200 RILs and their parents were planted at two locations in Beijing, Changping experimental station (N40°10′ and E116°14′) in 2009, and Shunyi (N40°13′ and E116°34′) experimental station from 2009 to 2011. The RILs were planted in rows 1.5 m long at 0.5 m intervals. Approximately 15 plants were planted in each row. Three replicates were conducted with a randomized complete block design.

A core panel of 110 RILs was selected randomly from the 200 RILs for genotyping and mapping analyses. The young leaves of each line of the core panel were collected, and total DNA of each of the parents and the 110 RILs were extracted using the CTAB method [50].

### SLAF library construction and high-throughput sequencing

The procedure used for SLAF library construction and high-throughput sequencing was performed as described by Sun et al. with minor modifications [16]. Firstly, a pilot experiment was performed based on the soybean reference genome sequences [16]. In this experiment, the enzymes and sizes of restriction fragments were evaluated using training data from the soybean reference genome sequences. Three criteria were considered: i) The number of SLAFs must be suitable for QTL mapping. ii) The SLAFs must be evenly distributed. iii) Repeated SLAFs must be avoided. To maintain the sequence depth uniformity of different fragments, a tight length range was selected (roughly 30-50 bp) and a pilot PCR amplification was performed to check the reduced representation library (RRL) features in this target length range. When non-specific amplified bands appeared on the gel, the pre-design step was repeated to produce a new scheme. The pilot experiment was to ensure that the SLAFs were evenly distributed across the soybean genome but that they did not appear in the repeated regions of the soybean genome. Based on the results of pilot experiment, the SLAF library was constructed as follows: genomic DNA of the 110 RIL was incubated at 37°C with *Mse*I [New England Biolabs, (NEB), Ipswich, MA, USA], T4 DNA ligase (NEB), ATP (NEB), and *Mse*I adapter. Restriction-ligation reactions were heat-inactivated at 65°C and then digested with the additional restriction enzyme *EcoR*I at 37°C. The PCR reaction was performed using diluted restriction-ligation samples, dNTP, Taq DNA polymerase (NEB) and *Mse*I primer containing barcode1. The PCR products were purified using an E.Z.N.A.® Cycle Pure Kit (Omega) and then pooled. The pooled samples were incubated with *Mse*I, T4-DNAligase, ATP and Solexa adapter at 37°C and then purified using a Quick Spin column (Qiagen, Hilden, Germany). The purified products were run on a 2% agarose gel. Fragments that were 500-550 bp (with indices and adaptors) in size were isolated using a Gel Extraction Kit (Qiagen, Hilden, Germany). The fragment products were subjected to PCR reaction with Phusion Master Mix (NEB) and Solexa amplification primer mix to add barcode2. The products that were 500-550 bp in size were gel-purified and diluted for pair-end sequencing. The pair-end sequencing was performed using an Illumina high-throughput sequencing platform (Illumina, Inc; San Diego, CA, U.S.).

### SLAF-seq data grouping and genotyping

The SLAF-seq data grouping and genotyping were performed as described in detail by Sun et al. [16]. All SLAF pair-end reads with clear index information were clustered based on sequence similarity. To reduce computational intensity, identical reads were merged together, and sequence similarity was detected using one-to-one alignment by BLAT [51] (-tileSize = 10 -stepSize = 5). Sequences with over 90% identity were grouped to one SLAF locus. Alleles were defined in each SLAF using the minor allele frequency (MAF) evaluation. Since soybean is a diploid species and one locus can only contain at most four SLAF tags, groups containing more than four tags were filtered out as repetitive SLAFs. SLAFs with sequence depth less than 213 were defined as low-depth SLAFs and were filtered out in the following analysis. Only groups with suitable depth and fewer than 4 seed tags were identified as high-quality SLAFs. SLAFs with 2-4 tags were identified as polymorphic SLAFs. The average sequence depths of SLAF markers were greater than 10-fold in parents and greater than 3-fold in progeny.

### High-density genetic map construction

After genotyping of the 110 RILs, 2-point linkage analysis was performed for efficient SLAFs. A high-density genetic map including 20 LGs was constructed using the Kosambi mapping function of the Joinmap v4.0 software [52]. The LOD threshold was set as default (3.0). The collinearity of LGs with the soybean reference genome was analyzed through aligning the sequence of each SLAF marker with genome sequences of Williams 82 using the BLASTN program from the National Center for Biotechnology Information (NCBI) [53].

### Isoflavone extraction and determination

The extraction and determination of isoflavones were performed according to the protocol described by Sun et al. [54]. First, approximately 20 g of mature seeds from each of the 110 RIL plants and their two parents were ground to a fine powder using a cyclone mill (Retsch ZM100, Rheinische, Germany). One hundred milligrams of this powder was added to a 10 ml glass tube preloaded with 5 ml of extract solution containing 0.1% (v/v) acetic acid and 70% (v/v) ethanol. The mixture was shaken for 24 h on a twist mixer (TM-300, ASONE, Japan). After centrifugation at 5,000 rpm for 10 min, the supernatant was filtered using the YMC Duo-filter (YMC Co., Kyoto Japan) with 0.2 μm pores. The resultant sample was stored at 4°C before use. The determination of isoflavones was performed with the High Performance Liquid Chromatography (HPLC) system (Agilent 1100, YMC-Pack, ODS-AM-303, 250 mm × 4.6 mm I.D., S-5 UM, 120 Å) using a 70-min linear gradient of 13% - 30% acetonitrile (v/v) in aqueous solution containing 0.1% acetic acid. The solvent flow rate was 1.0 ml min^−1^ and the UV absorption was measured at 260 nm. Column temperature was set at 35 °C and the injection volume was 10 μl. Identification and quantification of each isoflavone component was based on the standards of 12 isoflavone components provided by Dr. Akio Kikuchi (National Agricultural Research Center for Tohoku Region, Japan). The 12 isoflavone standards consisted of daidzein, glycitin, genistein, malonyldaidzin, malonylglycitin, malonylgenistin, acetyldaidzin, acetylglycitin, acetylgenistin, daidzein, glycitein and genistein. In this study, six major isoflavone components including daidzin, glycitin, genistin, malonyldaidzin, malonylglycitin, and malonylgenistin were detected in soybean seeds. The precise contents of these six isoflavone components were calculated with the formula described in detail by Sun et al. [54]. Other components were not quantified due to the very low concentrations. The total isoflavone content was designated as the sum of these six major isoflavone components.

### QTL analysis using high-density genetic map

The QTLs underlying the isoflavone content were identified using the ICIMapping v3.3 software [55]. Additive QTLs underlying the isoflavone content were identified by using the inclusive composite interval mapping (ICIM) method in the same software. The threshold of LOD scores for evaluating the statistical significance of QTL effects was determined using 1,000 permutations. Based on these permutations, a LOD score of 2.5 was used as a minimum to declare the presence of a QTL in a particular genomic region. The genes within QTLs were annotated and analyzed via the database of Phytozome v9.1 [56] and NCBI [53].

## Electronic supplementary material

Additional file 1: Figure S1: Heat map showing a matrix of pair-wise recombination values for SLAF markers along Gm20. The colors represent the strength of linkage in recombination values between all pairs of markers. The grey color indicates the lowest recombination scores, which suggest a strong linkage between markers. The red color represents the highest recombination scores, which suggest no linkage between markers. The yellow color represents middle recombination scores and some amount of linkage between markers. **Figure S2.** The collinearity map of Gm20. The yellow bar indicates the linkage group Gm20, while the blue bar indicates the corresponding chromosome of the soybean reference genome. The SLAF markers plus their location in centimorgan (cM) and kilo base pairs (Kb) with respect to the first marker in the LG are indicated on each side. **Figure S3.** Frequency distributions of six isoflavone components for the 110 RILs planted at Changping experimental station in 2009. **Figure S4.** Details of SLAF markers for 11 QTLs underlying individual and total isoflavone contents across various environments. The SLAF markers for these QTLs were indicated on the right side of LGs, while the genetic distance between adjacent SLAF markers were shown on the left side. **Table S1.** The correlation analysis of total isoflavone contents among four environments. **Table S2.** The characters of 41 QTLs associated with isoflavone content. (PDF 616 KB)

## Abbreviations

QTL: Quantitative trait locus
SLAF-seq: Specific length amplified fragment sequencing
NGS: Next generation sequencing
RIL: recombinant inbred line
LG: Linkage group
MAS: Marker-assisted selection
RFLP: Restriction fragment length polymorphism
AFLP: Amplified fragment length polymorphism
SSR: Simple sequence repeat
SNP: Single nucleotide polymorphism
LOD: Logarithm of odds
LD: Linkage disequilibrium
4CL: 4-coumarate: coenzyme A ligase
CHI: Chalcone isomerase
CHR: Chalcone reductase
IFS: Isoflavone synthase
OMT: O-methyltransferase
cM: Centimorgan
ANOVA: Analysis of variance
ICIM: Inclusive composite interval mapping.

## References

[1] **Soy Stats** [http://www.soystats.com]

[2] de Lumen BO (2005). Lunasin: a cancer-preventive soy peptide. Nutr Rev.

[3] Mateos-Aparicio I, Redondo Cuenca A, Villanueva-Suarez MJ, Zapata-Revilla MA (2008). Soybean, a promising health source. Nutr Hosp.

[4] Kato S, Sayama T, Fujii K, Yumoto S, Kono Y, Hwang TY, Kikuchi A, Takada Y, Tanaka Y, Shiraiwa T, Ishimoto M (2014). A major and stable QTL associated with seed weight in soybean across multiple environments and genetic backgrounds. Theor Appl Genet.

[5] Panthee DR, Pantalone VR, Sams CE, Saxton AM, West DR, Orf JH, Killam AS (2006). Quantitative trait loci controlling sulfur containing amino acids, methionine and cysteine, in soybean seeds. Theor Appl Genet.

[6] Cardinal AJ, Whetten R, Wang S, Auclair J, Hyten D, Cregan P, Bachlava E, Gillman J, Ramirez M, Dewey R, Upchurch G, Miranda L, Burton JW (2014). Mapping the low palmitate *fap1* mutation and validation of its effects in soybean oil and agronomic traits in three soybean populations. Theor Appl Genet.

[7] Tuyen DD, Lal SK, Xu DH (2010). Identification of a major QTL allele from wild soybean (*Glycine soja* Sieb. & Zucc.) for increasing alkaline salt tolerance in soybean. Theor Appl Genet.

[8] Concibido VC, Diers BW, Arelli PR (2004). A decade of QTL mapping for cyst nematode resistance in soybean. Crop Sci.

[9] Keim P, Diers BW, Olson TC, Shoemaker RC (1990). RFLP mapping in soybean: association between marker loci and variation in quantitative traits. Genetics.

[10] Gore MA, Hayes AJ, Jeong SC, Yue YG, Buss GR, Maroof S (2002). Mapping tightly linked genes controlling potyvirus infection at the *Rsv1* and *Rpv1* region in soybean. Genome.

[11] Hyten DL, Choi I-Y, Song Q, Specht JE, Carter TE, Shoemaker RC, Hwang E-Y KML, Cregan PB (2010). A high density integrated genetic linkage map of soybean and the development of a 1536 universal soy linkage panel for quantitative trait locus mapping. Crop Sci.

[12] Gutierrez-Gonzalez JJ, Vuong TD, Zhong R, Yu O, Lee JD, Shannon G, Ellersieck M, Nguyen HT, Sleper DA (2011). Major locus and other novel additive and epistatic loci involved in modulation of isoflavone concentration in soybean seeds. Theor Appl Genet.

[13] **Soybase** [http://www.soybase.org]

[14] Zou G, Zhai G, Feng Q, Yan S, Wang A, Zhao Q, Shao J, Zhang Z, Zou J, Han B, Tao Y (2012). Identification of QTLs for eight agronomically important traits using an ultra-high-density map based on SNPs generated from high-throughput sequencing in sorghum under contrasting photoperiods. J Exp Bot.

[15] Varshney RK, Nayak SN, May GD, Jackson SA (2009). Next-generation sequencing technologies and their implications for crop genetics and breeding. Trends biotech.

[16] Sun X, Liu D, Zhang X, Li W, Liu H, Hong W, Jiang C, Guan N, Ma C, Zeng H, Xu C, Song J, Huang L, Wang C, Shi J, Wang R, Zheng X, Lu C, Wang X, Zheng H (2013). SLAF-seq: an efficient method of large-scale *de novo* SNP discovery and genotyping using high-throughput sequencing. PLoS One.

[17] Chen S, Huang Z, Dai Y, Qin S, Gao Y, Zhang L, Chen J (2013). The development of 7E chromosome-specific molecular markers for Thinopyrum elongatum based on SLAF-seq technology. PLoS One.

[18] Zhang Y, Wang L, Xin H, Li D, Ma C, Ding X, Hong W, Zhang X (2013). Construction of a high-density genetic map for sesame based on large scale marker development by specific length amplified fragment (SLAF) sequencing. BMC Plant Biol.

[19] Lozovaya VV, Lygin AV, Zernova OV, Ulanov AV, Li S, Hartman GL, Widholm JM (2007). Modification of phenolic metabolism in soybean hairy roots through down regulation of chalcone synthase or isoflavone synthase. Planta.

[20] Shimada N, Sato S, Akashi T, Nakamura Y, Tabata S, Ayabe S, Aoki T (2007). Genome-wide analyses of the structural gene families involved in the legume-specific 5-deoxyisoflavonoid biosynthesis of *Lotus japonicus*. DNA Res.

[21] Novak K, Lisa L, Skrdleta V (2004). Rhizobial nod gene-inducing activity in pea nodulation mutants: dissociation of nodulation and flavonoid response. Physiol Plant.

[22] De Rijke E, Aardenburg L, Van Dijk J, Ariese F, Ernst WH, Gooijer C, Brinkman UA (2005). Changed isoflavone levels in red clover (*Trifolium pratense L.*) leaves with disturbed root nodulation in response to waterlogging. J Chem Ecol.

[23] Ferrer JL, Austin MB, Stewart C, Noel JP (2008). Structure and function of enzymes involved in the biosynthesis of phenylpropanoids. Plant Physiol Biochem.

[24] Joung JY, Kasthuri GM, Park JY, Kang WJ, Kim HS, Yoon BS, Joung H, Jeon JH (2003). An overexpression of chalcone reductase of *Pueraria montana* var. *lobata* alters biosynthesis of anthocyanin and 5'-deoxyflavonoids in transgenic tobacco. Biochem Biophys Res Commun.

[25] Sarkar FH, Li Y (2003). Soy isoflavones and cancer prevention. Cancer Invest.

[26] Cornwell T, Cohick W, Raskin I (2004). Dietary phytoestrogens and health. Phytochemistry.

[27] Dixon RA (2004). Phytoestrogens. Annu Rev Plant Biol.

[28] Ali AA, Velasquez MT, Hansen CT, Mohamed AI, Bhathena SJ (2005). Modulation of carbohydrate metabolism and peptide hormones by soybean isoflavones and probiotics in obesity and diabetes. J Nutr Biochem.

[29] Cogolludo A, Frazziano G, Briones AM, Cobeno L, Moreno L, Lodi F, Salaices M, Tamargo J, Perez-Vizcaino F (2007). The dietary flavonoid quercetin activates BKCa currents in coronary arteries via production of H_2_O_2_. Role in vasodilatation. Cardiovasc Res.

[30] Moore AB, Castro L, Yu L, Zheng X, Di X, Sifre MI, Kissling GE, Newbold RR, Bortner CD, Dixon D (2007). Stimulatory and inhibitory effects of genistein on human uterine leiomyoma cell proliferation are influenced by the concentration. Hum Reprod.

[31] Di X, Yu L, Moore AB, Castro L, Zheng X, Hermon T, Dixon D (2008). A low concentration of genistein induces estrogen receptor-alpha and insulin-like growth factor-I receptor interactions and proliferation in uterine leiomyoma cells. Hum Reprod.

[32] Jung W, Yu O, Lau SM, O'Keefe DP, Odell J, Fader G, McGonigle B (2000). Identification and expression of isoflavone synthase, the key enzyme for biosynthesis of isoflavones in legumes. Nat Biotechnol.

[33] Dhaubhadel S, McGarvey BD, Williams R, Gijzen M (2003). Isoflavonoid biosynthesis and accumulation in developing soybean seeds. Plant Mol Biol.

[34] Deavours BE, Dixon RA (2005). Metabolic engineering of isoflavonoid biosynthesis in alfalfa. Plant Physiol.

[35] Wang X (2010). Structure, function, and engineering of enzymes in isoflavonoid biosynthesis. Funct Integr Genomics.

[36] Yi J, Derynck MR, Li X, Telmer P, Marsolais F, Dhaubhadel S (2010). A single-repeat MYB transcription factor, GmMYB176, regulates *CHS8* gene expression and affects isoflavonoid biosynthesis in soybean. Plant J.

[37] Primomo VS, Poysab V, Ablettc GR, Jacksond CJ, Gijzene M, Rajcan I (2005). Mapping QTL for individual and total isoflavone content in soybean seeds. Crop Sci.

[38] Gutierrez-Gonzalez JJ, Wu X, Zhang J, Lee JD, Ellersieck M, Shannon JG, Yu O, Nguyen HT, Sleper DA (2009). Genetic control of soybean seed isoflavone content: importance of statistical model and epistasis in complex traits. Theor Appl Genet.

[39] Zeng G, Li D, Han Y, Teng W, Wang J, Qiu L, Li W (2009). Identification of QTL underlying isoflavone contents in soybean seeds among multiple environments. Theor Appl Genet.

[40] Gutierrez-Gonzalez JJ, Wu X, Gillman JD, Lee JD, Zhong R, Yu O, Shannon G, Ellersieck M, Nguyen HT, Sleper DA (2010). Intricate environment-modulated genetic networks control isoflavone accumulation in soybean seeds. BMC Plant Biol.

[41] Yang K, Moon JK, Jeong N, Chun HK, Kang ST, Back K, Jeong SC (2011). Novel major quantitative trait loci regulating the content of isoflavone in soybean seeds. Genes & Genomics.

[42] Kassem MA, Meksem K, Iqbal MJ, Njiti VN, Banz WJ, Winters TA, Wood A, Lightfoot DA (2004). Definition of soybean genomic regions that control seed phytoestrogen amounts. J Biomed Biotechnol.

[43] Meksem K, Njiti VN, Banz WJ, Iqbal MJ, Kassem MM, Hyten DL, Yuang J, Winters TA, Lightfoot DA (2001). Genomic regions that underlie soybean seed isoflavone content. J Biomed Biotechnol.

[44] Hisano H, Sato S, Isobe S, Sasamoto S, Wada T, Matsuno A, Fujishiro T, Yamada M, Nakayama S, Nakamura Y, Watanabe S, Harada K, Tabata S (2007). Characterization of the soybean genome using EST-derived microsatellite markers. DNA Res.

[45] Zhang J, Ge Y, Han F, Li B, Yan S, Sun J, Wang L (2014). Isoflavone content of soybean cultivars from maturity group 0 to VI grown in northern and southern China. J Am Oil Chem Soc.

[46] Lam HM, Xu X, Liu X, Chen W, Yang G, Wong FL, Li MW, He W, Qin N, Wang B, Li J, Jian M, Wang J, Shao G, Sun SS, Zhang G (2010). Resequencing of 31 wild and cultivated soybean genomes identifies patterns of genetic diversity and selection. Nature Genet.

[47] Hyten DL, Song Q, Choi IY, Yoon MS, Specht JE, Matukumalli LK, Nelson RL, Shoemaker RC, Young ND, Cregan PB (2008). High-throughput genotyping with the GoldenGate assay in the complex genome of soybean. Theor Appl Genet.

[48] Smallwood CJ (2012). Detection of quantitative trait loci for marker-assisted selection of soybean isoflavone genistein. Masters Theses.

[49] Du H, Huang Y, Tang Y (2010). Genetic and metabolic engineering of isoflavonoid biosynthesis. Appl Microbiol Biotechnol.

[50] Doyle JJ, Doyle JL (1990). Isolation of plant DNA from fresh tissue. Focus.

[51] Kent WJ (2002). BLAT-the BLAST-like alignment tool. Genome Res.

[52] Stam P (1993). Construction of integrated genetic linkage maps by means of a new computer package: Join Map. Plant J.

[53] **National Center for Biotechnology Information** [http://www.ncbi.nlm.nih.gov]

[54] Sun J, Sun B, Han F, Yan S, Yang H, Akio K (2011). Rapid HPLC method for determination of 12 isoflavone components in soybean seeds. Agri Sci China.

[55] Li H, Ribaut JM, Li Z, Wang J (2008). Inclusive composite interval mapping (ICIM) for digenic epistasis of quantitative traits in biparental populations. Theor Appl Genet.

[56] **Joint Genome Institute, USDOE** [http://www.phytozome.net]

