# Supplementary material for: Construction of a high-density genetic map based on large-scale markers developed by specific length amplified fragment sequencing (SLAF-seq) and its application to QTL analysis for isoflavone content in Glycine max
Source: BMC Genomics. 2014 Dec 10;15(1):1086. doi: 10.1186/1471-2164-15-1086 (PMC4320444; doi:10.1186/1471-2164-15-1086)
Supplement: Supplementary file 1 — Additional file 1: Figure S1: Heat map showing a matrix of pair-wise recombination values for SLAF markers along Gm20. The colors represent the strength of linkage in recombination values between all pairs of markers. The grey color indicates the lowest recombination scores, which suggest a strong linkage between markers. The red color represents the highest recombination scores, which suggest no linkage between markers. The yellow color represents middle recombination scores and some amount of linkage between markers. Figure S2. The collinearity map of Gm20. The yellow bar indicates the linkage group Gm20, while the blue bar indicates the corresponding chromosome of the soybean reference genome. The SLAF markers plus their location in centimorgan (cM) and kilo base pairs (Kb) with respect to the first marker in the LG are indicated on each side. Figure S3. Frequency distributions of six isoflavone components for the 110 RILs planted at Changping experimental station in 2009. Figure S4. Details of SLAF markers for 11 QTLs underlying individual and total isoflavone contents across various environments. The SLAF markers for these QTLs were indicated on the right side of LGs, while the genetic distance between adjacent SLAF markers were shown on the left side. Table S1. The correlation analysis of total isoflavone contents among four environments. Table S2. The characters of 41 QTLs associated with isoflavone content. (PDF 616 KB) [file 12864_2014_6981_MOESM1_ESM.pdf]

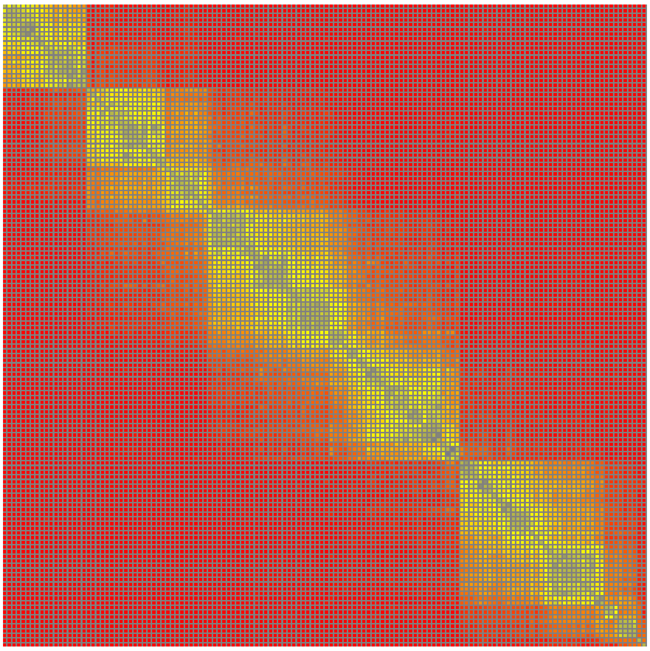

Supplementary Figure S1 Heat map showing a matrix of pair-wise recombination values for SLAF markers along Gm20. The colors represent strength of linkage in recombination values between all pairs of markers. The grey color indicates the lowest recombination scores, which suggests a strong linkage between markers. The red color represents the highest recombination scores, which suggests no linkage between markers. The yellow color represents middle recombination scores and some amount of linkage between markers.

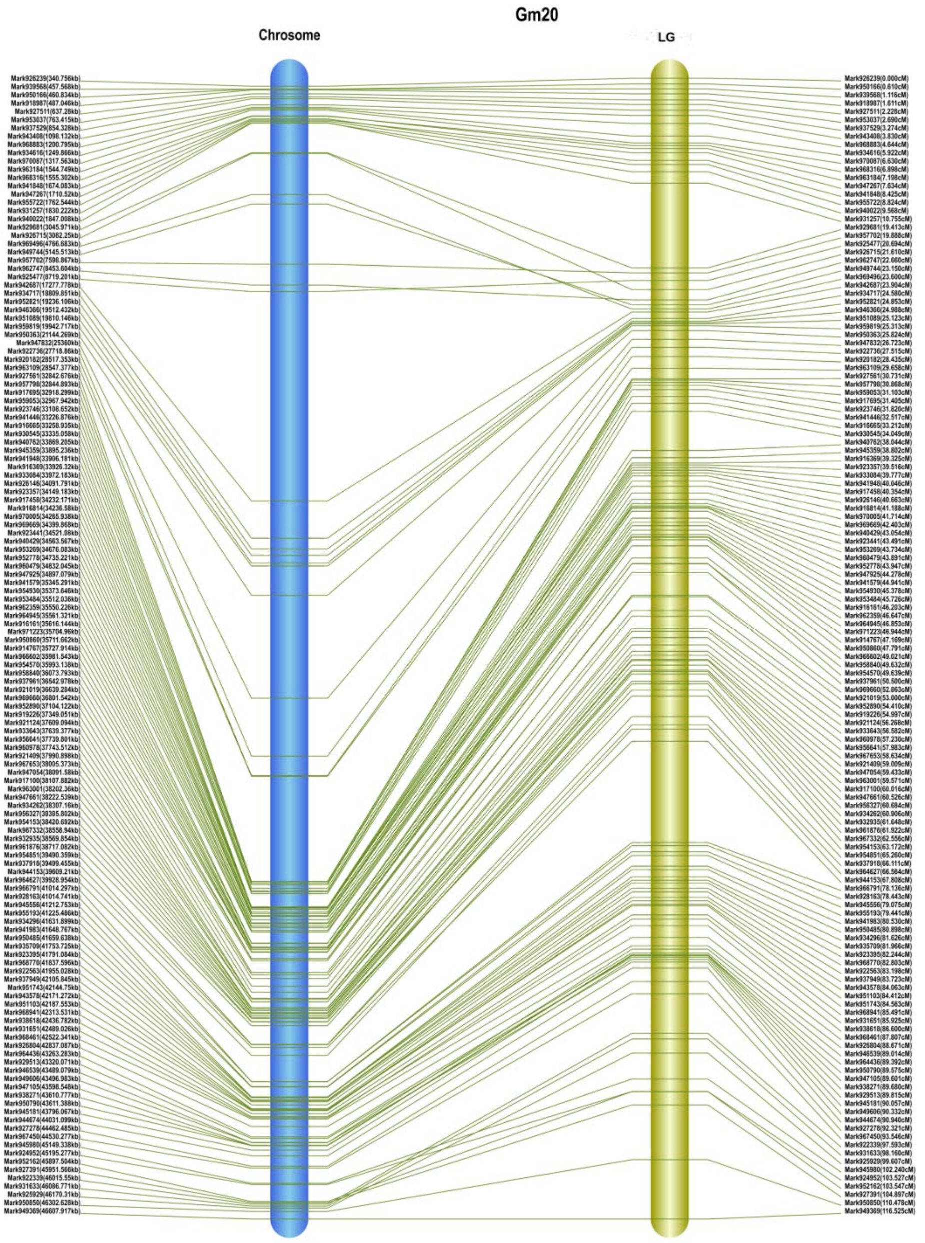

Supplementary Figure S2 The collinearity map of Gm20. The yellow bar indicates the linkage group Gm20, while the blue bar indicates the corresponding chromosome of the soybean reference genome. The SLAF markers plus their location in centimorgan (cM) and kilo base pairs (Kb) with respect to the first marker in the LG are indicated on each side.

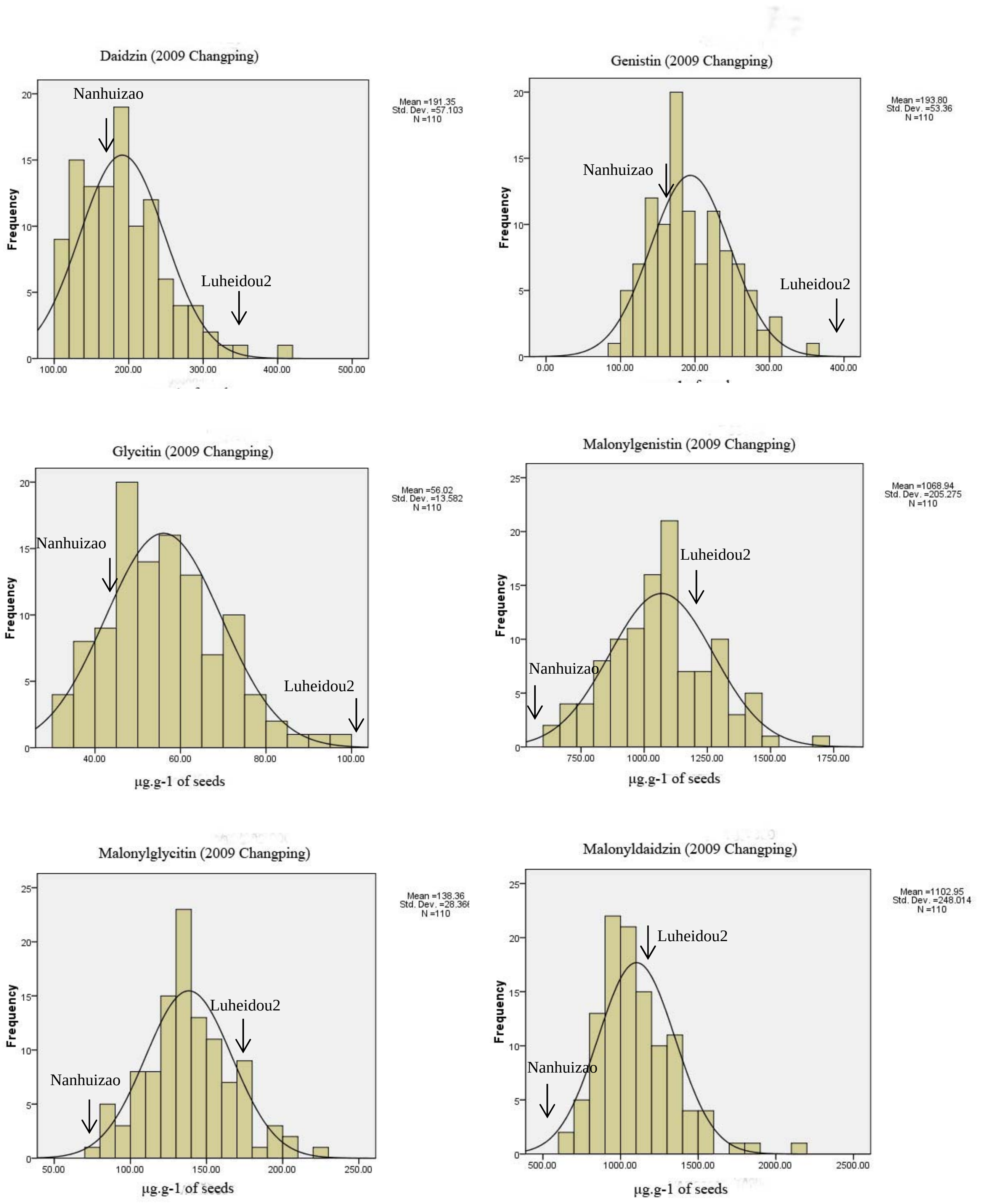

Supplementary Figure S3 Frequency distributions of six isoflavone components for the 110 RILs planted at Changping experimental station in 2009. The six isoflavone components include daidzin (upper left panel), genistin (upper right panel), glycitin (middle left panel), malonylgenistin (middle right panel), malonylglycitin (lower left panel), and malonyldaidzin (lower right panel). The arrows indicate the contents of corresponding isoflavone components for two parents of the RILs (*cv*. Luheidou 2 and Nanhuizao).

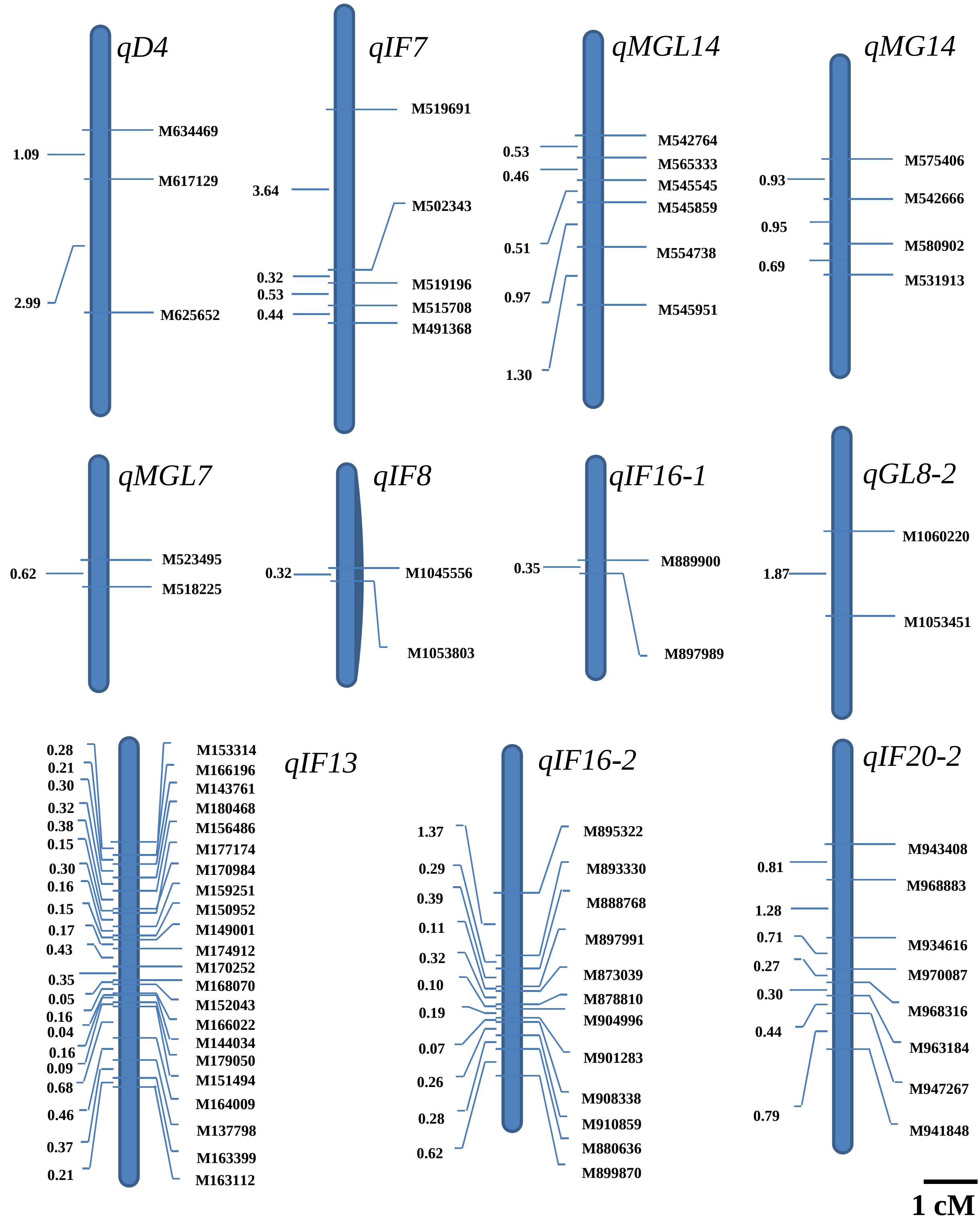

Supplementary Figure S4 Details of SLAF markers for 11 QTLs underlying individual and total isoflavone contents across various environments. The SLAF markers for these QTLs were indicated on the right side of LGs, while the genetic distance between adjacent SLAF markers were shown on the left side.

Supplementary Table S1 The correlation analysis of total isoflavones among four environments

	Total isoflavones _E1	Total isoflavones_E2	Total isoflavones_E3
Total isoflavones_E2	0.60**		
Total isoflovones_E3	0.57**	0.78 **	
Total isoflavones_E4	0.60**	0.71 **	0.70 **

E1-E4 indicate the four environments used in this study: E1 indicates that the RILs were planted at Changping Experimental Station in 2009, E2 indicates that the RILs were planted at Shunyi Experimental Station in 2009, E3 indicates that the RILs were planted at Shunyi Experimental Station in 2010, and the E4 indicates that the RILs were planted at Shunyi Experimental Station in 2011.

**indicates that the correlation is significant at the 0.01 level ($P < 0.01$).

Supplementary Table S1 The characters of 41 QTLs associated with individual and total isoflavone contents

QTL[1]	Effect[2]	Chr.[3]	Interval[4]	IC[5]	LOD[6]	PVE[7]	ADD[8]	Isoflavone biosynthetic enzyme-encoding gene	Previous reported
qG1	G-E2	1	M454526-M417340	6.59-7.53	4.30	8.90	-20.00	*Glyma01g01350 (4CL6)*	
qTOT2	TOT-E2	2	M812331-M781433	73.98-76.47	3.57	3.48	122.00		TOT [1]
qMD3	MD-E2	3	M702174-M696198	15.79-16.31	3.51	8.73	92.60		
qMG3	MG-E2		M651981-M679553	1.98-2.09	4.68	5.44	56.80		
qD4	D-E3,E4	4	M634469-M625652	14.67-18.75	6.98	16.00	23.40		GE,TOT [2]
qMG5-1	MG-E2	5	M254318-M241975	20.35-21.02	3.55	4.61	-52.00		DE [3,4]
qMG5-2	MG-E2		M267738-M266007	98.91-99.03	6.13	7.14	66.50		DE,GE,TOT,GLE [1,2,5-8]
qGL5	GL-E3		M270783-M251548	41.69-42.05	4.79	9.68	5.79		GE, GLE [5,7]
qTOT6-1	TOT-E3	6	M390668-M377794	16.81-17.25	2.81	3.50	148.00		
qTOT6-2	TOT-E2		M352799-M386087	74.83-75.03	24.4	39.00	-400.00		
qIF6	MGL-E1; G-E2		M393180-M366472	58.89-60.39	5.29	8.63	7.07		
qGL7	GL-E2	7	M504981-M495232	87.84-88.14	8.61	10.90	6.66	*Glyma07g32330 (IFS1)*	DE, GE, TOT [6]
qMD7	MD-E3		M488485-M522236	0.00-0.21	6.13	13.00	-101.00		
qMGL7	MGL-E2,E3		M523495-M518225	3.62-4.24	4.59	13.30	-23.00		
qIF7	G-E2,E3; MD-E2,E3 MG-E2,E3;TOT-E2,E3		M519691-M491368	33.21-38.13	8.17	14.30	137.00		DE,GE,GLE,TOT [7]
qGL8-1	GL-E2	8	M1053623-M1029773	26.74-28.09	12.00	15.20	-7.80		DE [7]
qGL8-2	GL-E2,E4		M1060220-M1053451	30.48-32.34	12.40	28.00	8.98		DE [7]
qGL8-3	GL-E1		M1067959-M1052450	53.86-54.29	4.41	18.50	-5.80		
qIF8	MG-E1,E4; TOT-E4		M1045556-M1053803	1.44-1.76	5.73	16.90	144.00		

QTL[1]	Effect[2]	Chr.[3]	Interval[4]	IC[5]	LOD[6]	PVE[7]	ADD[8]	Isoflavone biosynthetic enzyme-encoding gene	Previous reported
qIF9	G-E1;TOT-E1	9	M200687-M218331	87.75-95.10	3.77	14.40	117.00		DE,GE,GLE,TOT [9]
qTOT10	TOT-E4	10	M323025-M308581	63.19-64.16	2.66	8.35	151.00		
qD11	D-E2		M991205-M987236	52.83-61.54	2.81	7.74	17.00		DE, GE, GLE, TOT [1,3-5]
qGL11	GL-E2	11	M974688-M1014283	76.79-77.40	3.91	4.94	4.70		GLE [5]
qIF11	GL-E2; TOT-E2		M1002626-M990325	6.36-15.95	8.38	9.28	96.50		
qMG12	MG-E4	12	M864816-M843903	11.90-12.08	3.44	8.44	-80.00		
qG13	G-E4		M173060-M134040	65.83-68.08	6.12	16.10	-28.00		DE [7]
qIF13	D-E2,E3,E4; G-E3; MG-E3; MD-E4	13	M153314-M163112	45.77-51.16	4.91	12.50	-44.00	*Glyma13g24200 (IFS2)* *Glyma13g24210 (OMT)*	DE [7]
qG14	G-E3		M584735-M574287	13.80-15.43	11.3	27.40	67.10		
qMG14	MG-E1,E3		M575406-M531913	0.00-2.57	3.50	9.54	83.10	*Glyma14g00870 (CHR)*	
qMGL14	MGL-E1,E3	14	M542764-M545951	6.83-10.60	4.00	8.61	9.96		
qIF14-1	MG-E4;TOT-E4		M545951-M579552	10.60-12.62	5.31	15.80	154.00		
qIF14-2	GL-E3;G-E3		M535325-M589440	20.51-21.14	5.34	11.50	-21.00		
qIF16-1	D-E2,E3; TOT-E2	16	M889900-M897989	20.85-21.19	3.96	8.56	-49.00		DE,TOT [2]
qIF16-2	MD-E1,E3,E4; D-E4		M895322-M899870	59.53-63.53	5.12	15.10	-79.00		
qIF17-1	MG-E2;TOT-E2	17	M1082607-M1109374	82.02-91.26	5.01	5.73	-111.00	*Glyma17g34435 (CHI)*	GLE [6]
qIF17-2	GL-E3;MGL-E3		M1095927-M1100661	16.07-22.25	3.80	6.93	6.87	*Glyma17g07190 (4CL1)* *Glyma17g07170 (4CL1)* *Glyma17g07190 (4CL1)*	GE , GLE [6,9]
qG18	G-E2	18	M69732-M107260	56.24-57.17	7.63	18.50	29.10		TOT [10]
qMGL19	MGL-E1	19	M749182-M727854	99.74-100.07	5.08	12.70	-10.00		

QTL[1]	Effect[2]	Chr.[3]	Interval[4]	IC[5]	LOD[6]	PVE[7]	ADD[8]	Isoflavone biosynthetic enzyme-encoding gene	Previous reported
qGL20	GL-E4		M937949-M943578	83.72-84.06	4.29	15.20	5.04	*Glyma20g33370 (4CL5)*	
								Glyma20g38580 (CHI3)	
qIF20-1	G-E4;MD-E2;TOT-E3		M945980-M950850	2.24-10.48	4.80	10.30	110.00	*Glyma20g38570 (CHI2)*	
								Glyma20g38560 (CHI1)	
	D-E1,E2,E3,E4	20							
	G-E2,E3,E4								
qIF20-2	MD-E1,E2,E3,E4		M943408-M941848	3.83-8.43	9.11	19.60	117.00		
	MG-E2,E3								
	TOT-E1,E2,E3								

[1]The name of the QTL, is a composite of the influenced trait: genistin (G), daidzin (D), glycitin (GL), malonyldaidzin (MD), ma1ony1genistin (MG), ma1ony1g1ycitin (MGL) and total of isoflavones (TOT) followed by the chromosome number. For QTLs underlying the contents of multiple isoflavone components, the name is a composite of isoflavones (IF) followed by the chromosome number.

[2] The Effect of QTL is composite of the particular isoflavone component followed by the specific environments. It represents the particular isoflavone components [i. e., genistin (G), daidzin (D), glycitin (GL), malonyldaidzin (MD), ma1ony1genistin (MG), ma1ony1g1ycitin (MGL)] and total isoflavones (TOT) that are controlled by this QTL in specific environments [i. e., E1 (2009 at Changping), E2 (2009 at Shunyi), E3 (2010 at Shunyi), and E4 (2011 at Shunyi)].

[3]Chr indicates chromosome.

[4]Interval indicates confidence intervals between two SLAF markers.

[5]IC indicates the interval of confidence in centimorgan.

[6]LOD indicates the logarithm of odds score.

[7]PVE indicates the phenotypic variance explained by individual QTL.

[8]ADD indicates the additive effect value.

The LOD scores, PVE and additive values are presented as mean values for QTLs with multiple effects.

Reference

1. Yoshikawa T, Okumoto Y, Ogata D, Sayama T, Teraishi M, Terai M, TodaT, Yamada K, Yagasaki K, Yamada N, Tsukiyama T, Yamada T, Tanisaka T: **Transgressive segregation of isoflavone contents under the control of four QTLs in a cross between distantly related soybean varieties.** *Breeding Sci* 2010, **60**:243-254.
2. Gutierrez-Gonzalez JJ, Wu X, Gillman JD, Lee JD, Zhong R, Yu O, Shannon G, Ellersieck M, Nguyen HT, Sleper DA: **Intricate environment-modulated genetic networks control isoflavone accumulation in soybean seeds.** *BMC Plant Biol* 2010, **10**:105.
3. Kassem MA, Meksem K, Iqbal MJ, Njiti VN, Banz WJ, Winters TA, Wood A, Lightfoot DA: **Definition of soybean genomic regions that control seed phytoestrogen amounts.** *J Biomed Biotech* 2004, **1**:52-60.
4. Meksem K, Njiti VN, Banz WJ, Iqbal MJ, Kassem MM, Hyten DL, Yuang J, Winters TA, Lightfoot DA: **Genomic regions that underlie soybean seed isoflavone content.** *J Biomed Biotech* 2001, **1**(1):38-44.
5. Gutierrez-Gonzalez JJ, Vuong TD, Zhong R, Yu O, Lee JD, Shannon G, Ellersieck M, Nguyen HT, Sleper DA: **Major locus and other novel additive and epistatic loci involved in modulation of isoflavone concentration in soybean seeds.** *Theor Appl Genet* 2011, **123**(8):1375-1385.
6. Gutierrez-Gonzalez JJ, Wu X, Zhang J, Lee JD, Ellersieck M, Shannon JG, Yu O, Nguyen HT, Sleper DA: **Genetic control of soybean seed isoflavone content: importance of statistical model and epistasis in complex traits.** *Theor App Genet* 2009, **119**(6):1069-1083.
7. Primomo VS, Poysab V, Ablettc GR, Jacksond CJ, Gijzene M, Rajcan I: **Mapping QTL for individual and total isoflavone content in soybean seeds.** *Crop Sci* 2005, **45**(6): 2454-2464.
8. Yang K, Moon JK, Jeong N, Chun HK, Kang ST, Back K, Jeong SC: **Novel major quantitative trait loci regulating the content of isoflavone in soybean seeds.**

Genes & Genomics 2011, **33**(6):685-692.

9. Smallwood CJ: **Detection of Quantitative Trait Loci for Marker-Assisted Selection of Soybean Isoflavone Genistein.** *Masters Theses*. the University of Tennessee; 2012.

10. Zeng G, Li D, Han Y, Teng W, Wang J, Qiu L, Li W: **Identification of QTL underlying isoflavone contents in soybean seeds among multiple environments.** *Theor App Genet* 2009, **118**(8):1455-1463.
